# Artificial Intelligence Its Uses and Application in Pediatric Dentistry: A Review

**DOI:** 10.3390/biomedicines11030788

**Published:** 2023-03-05

**Authors:** Satish Vishwanathaiah, Hytham N. Fageeh, Sanjeev B. Khanagar, Prabhadevi C. Maganur

**Affiliations:** 1 Department of Preventive Dental Sciences, Division of Pediatric Dentistry, College of Dentistry, Jazan University, Jazan 45142, Saudi Arabia; 2 Department of Preventive Dental Sciences, Division of Periodontics, College of Dentistry, Jazan University, Jazan 45142, Saudi Arabia; 3 Preventive Dental Science Department, College of Dentistry, King Saud Bin Abdulaziz University for Health Sciences, Riyadh 11426, Saudi Arabia; 4 King Abdullah International Medical Research Centre, Ministry of National Guard Health Affairs, Riyadh 11481, Saudi Arabia

**Keywords:** artificial intelligence, machine learning, convolutional neural network, deep learning, pediatric dentistry

## Abstract

In the global epidemic era, oral problems significantly impact a major population of children. The key to a child’s optimal health is early diagnosis, prevention, and treatment of these disorders. In recent years, the field of artificial intelligence (AI) has seen tremendous pace and progress. As a result, AI’s infiltration is witnessed even in those areas that were traditionally thought to be best left to human specialists. The ultimate ability to improve patient care and make precise diagnoses of illnesses has revolutionized the world of healthcare. In the field of dentistry, the competence to execute treatment measures while still providing appropriate patient behavior counseling is in high demand, particularly in the field of pediatric dental care. As a result, we decided to conduct this review specifically to examine the applications of AI models in pediatric dentistry. A comprehensive search of the subjects was done using a wide range of databases to look for studies that have been published in peer-reviewed journals from its inception until 31 December 2022. After the application of the criteria, only 25 of the 351 articles were taken into consideration for this review. According to the literature, AI is frequently used in pediatric dentistry for the purpose of making an accurate diagnosis and assisting clinicians, dentists, and pediatric dentists in clinical decision making, developing preventive strategies, and establishing an appropriate treatment plan.

## 1. Introduction

The idea of “Artificial Intelligence” (AI) was conceived in the year 1943, but the term was coined by John McCarthy at a conference in the year 1956, and the concept revolved around manufacturing machines that could replicate the tasks done by mankind [1,2]. AI is a complex term to define, but in the larger sense, it is a machine algorithm that can reason out and execute cognitive functions [3,4]. Nowadays, instead of the hand-crafted guidelines used in the past, machine learning (ML) and deep learning (DL) are two major branches of AI that are used in medicine [5]. ML develops algorithms and statistical models using computers to improve cognition and understanding [6]. It includes training algorithms on big datasets to detect patterns and utilize these patterns to forecast or decide upon fresh data [7]. On the other hand, DL is a subset of machine learning that uses artificial neural networks to mimic the learning process of the human brain [8]. They are trained using large amounts of data and algorithms and are more accurate [5]. One class of DL is the Artificial Neural Network (ANN), which comprises tiny communicating units known as neurons that are arranged in layers; deep learning is nothing but an ANN with multiple hidden layers. A subclass of ANN, convolutional neural networks (CNN), is predominantly used in dentistry and general medicine [6,8,9]. Using a subsampling layer similar to a multilayer perceptron and fully linked layers, CNNs, a particular class of DL, are able to completely fill an image with cells from the visual cortex [10].

CNNs can identify anatomical structures, detect dental caries, and replace cone-beam computer tomography (CBCT) in the field of endodontics because CBCT requires high doses of radiation. They also play an indispensable role in oral pathology [9,11]. There are different types of neural networks for a wide range of applications. Some examples are the regional-based convolutional network (R CNN) [12], the Faster region proposal with CNN features (fast R-CNN) [13], faster R-CNN [14], and the mask R-CNN [15].

AI is a recent technological advance that has swiftly acquired traction in the field of science and technology. AI heavily relies upon imaging, which thrives as a cornerstone for dentistry to a large extent. AI is highly beneficial in assessing and monitoring a patient’s health continuously, understanding the long-term effects of a drug, and knowing any health-related risk beforehand [8,9,16]. AI has the potential to completely eliminate the long hours invested by dental professionals. Additionally, it is feasible to improve people’s health at lower costs, provide customized, preventative, and predictive dentistry, and integrate healthcare for everyone. Above all, AI has the potential to increase the standards of dental care, fine-tune the accuracy and effectiveness of diagnosis, come up with better visuals for treatment, simulate results, and predict oral diseases and health [6,8,9,16]. AI models have also gained attention for their use as ancillary tools, increasing the precision and accuracy of diagnoses. AI technology has been widely applied in the field of medical sciences and has demonstrated excellent performance in a variety of tasks related to patient care, including disease diagnoses and identification of a patient’s risk for developing a disease, among many other tasks [17,18,19].

A major reason to provide this review was the speedy upsurge in new studies related to neural networks in the field of dentistry. In pediatric dentistry, AI has many promising applications that might change pediatric practice in the coming years. Thus, the aim of this research was to explore the multitudinous possibilities of using AI in pediatric dentistry.

## 2. Materials and Methods

### 2.1. Search Strategy

Various papers containing data related to the use of AI models in pedodontics were fetched using the most recognized electronic databases, including PubMed, Google Scholar, Web of Science, Scopus, and Saudi Digital. A search was carried out for original research articles published from inception until 31 December 2022, in peer-reviewed journals.

Electronic databases made a thorough search of the articles using various medical subheadings (MeSH keywords) such as Artificial Intelligence, AI, automated diagnostics, computer-assisted diagnosis, digital diagnostics, supervised learning, pedodontics, pediatric dentistry, growing children, unsupervised learning, diagnosis, prognosis, prediction, deep learning, machine learning, AI, and convolutional neural networks. These key terms were combined in the advanced search using the Boolean operators AND/OR alongside the use of English language filters. After checking the references of the papers retrieved via the electronic search, a manual search for articles was conducted concurrently.

At this juncture, after selecting articles based on their titles and abstracts, 351 relevant articles were retrieved. After removing 242 duplicate articles, only 106 papers met the qualifying requirements for inclusion in this review article.

### 2.2. Eligibility Criteria and Study Selection

**Figure 1:**
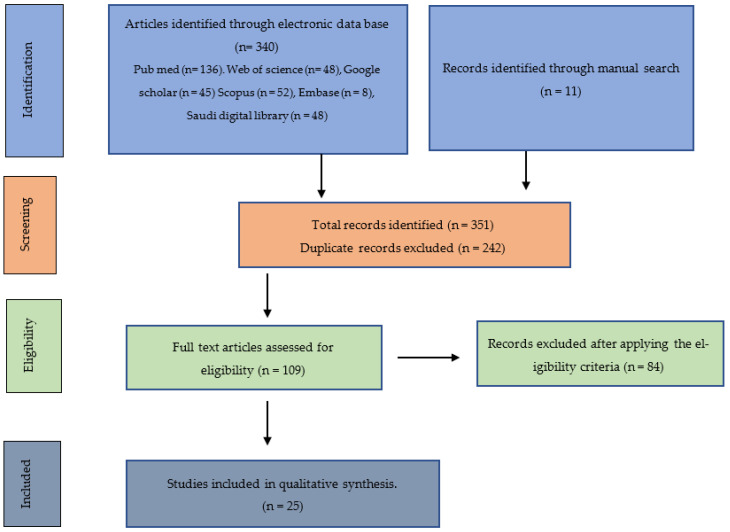
Flow chart for screening and selecting articles.

This current review includes original research publications on the application of AI models in pedodontics and pediatric dentistry. Articles without complete texts, narrative reviews, scoping reviews, letters to the editor, opinion letters, case reports, brief communications, conference proceedings, and non-English language articles (84 articles) were all omitted (Figure 1). Finally, only 25 papers met the qualifying requirements [3,7,10,20,21,22,23,24,25,26,27,28,29,30,31,32,33,34,35,36,37,38,39,40,41].

## 3. Results

### 3.1. Descriptive Analysis of the Included Studies

Table 1 describes the final analysis of the included studies. Author details, publication year, algorithm architecture used, the objective of the study, the outcome of the study, and the author’s observation were the details recorded for each study.

A summary of AI models designed for different diagnostic tasks, which includes AI technique/algorithm, architecture, diagnostic tasks, the functionality of the AI model, and Input features, is presented in Table 2.

**Figure 2:**
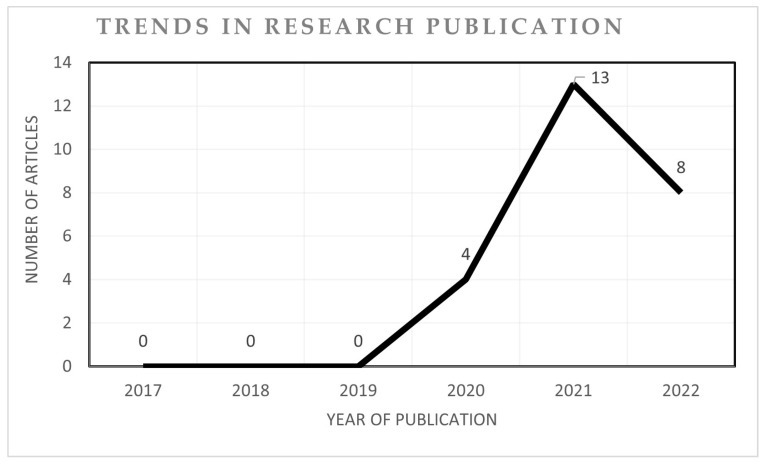
Trends in Research Publication.

The research trend shows an increase in the number of research publications over the last decades, and, in the last two years, the number of articles reported on the application of AI models in pedodontics has rapidly increased (Figure 2).

### 3.2. Study Characteristics

**Figure 3:**
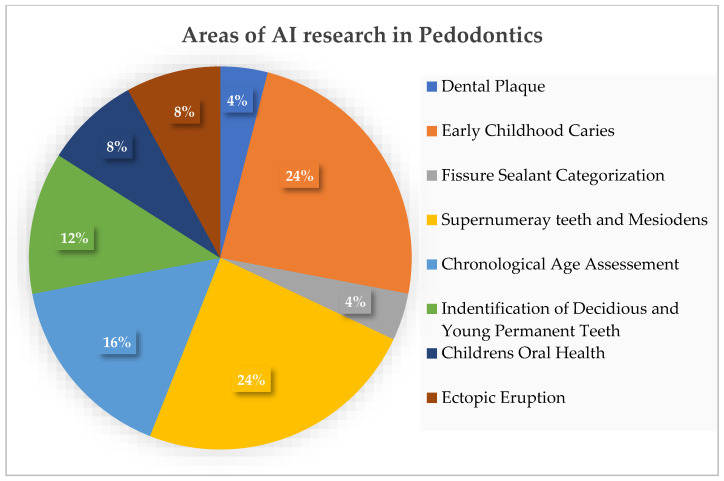
Areas of AI research in Pedodontics.

AI models developed for application in pedodontics have mainly focused on: dental plaque on primary teeth (*n* = 1) [21], ECC (*n* = 6) [25,26,27,28,29,30], fissure sealant categorization (*n* = 1) [31], mesiodens and supernumerary tooth identification (*n* = 6) [3,10,22,23,24,41], chronological age assessment (*n* = 4) [32,33,37,38], identification of deciduous and young permanent teeth (*n* = 3) [34,35,39], children’s oral Health (*n* = 2) [7,20], and ectopic eruption (*n* = 2) [39,40] (Figure 3).

### 3.3. Outcome Measures

The major outcome measures reported in the included studies were accuracy, sensitivity, specificity, Area Under the Curve (AUC), Mean Absolute Error (MAE), Root Mean Square Error (RMSE), F1 scores, Positive Predictive Value (PPV), Intersection Over Union (IoU), Mean Average Precision(mAP), Mean Average Recall (mAR), Area under the receiver operating characteristic (AUROC), and Area under the free response ROC curve (FROC).

## 4. Discussion

It is highly feasible to guarantee the top-most dental treatment by employing AI systems as a supplementary aid to dentists. This way, we can expect better prediction of treatment results and improvements in diagnosis precision and treatment planning. While deep learning primarily assists dentists with diagnosis, AI claims to improve accuracy and precision besides increasing the productivity of the dentist. AI applications have found their use in every field of endodontics, including root fractures, periapical lesions, dental and root caries, stem cell viability, the anatomy of the root canal system, and more [16]. As and when data availability became easier, AI started playing a vital role in proving its benefits in various pediatric dental procedures. CNN models are utilized in pediatric dentistry, in particular, for quicker and more accurate diagnosis. Such usage, in turn, encourages patients to display better cooperation with their dental practitioners, thereby increasing the success rate of dental treatment.

### 4.1. Dental Plaque

Dental plaque is a condition personified by a bacterial community sticking to the surface of the teeth, predominantly at the margins of the gingiva, including the interproximal sites [21]. Detecting their presence is quite tricky, even for an experienced dentist, especially when they are present in meager quantities, as distinguishing between plaque and the tooth is a challenge. Until recently, clinicians have been using an explorer or a disclosing solution to mark the infected area, but these methods are not only unwieldy but also rather inconvenient. Other disadvantages, more on the aesthetic side, include their unpalatable taste and retained stains on the lips and oral membrane [21]. Though other methods involving digital imaging analysis and autofluorescence spectroscopy are in use, they do have issues with cost and technology [42,43,44]. The digital camera era, together with the availability of image analysis software, was the first successful imaging system that measured the total plaque-affected area if present [45,46].

However, now, AI model-based deep learning techniques to identify plaque-affected primary teeth, a first-of-its-kind study, are under research. You, W. et al. [21], in their study, have successfully delivered AI systems (CNN framework) that were trained on 886 tooth photos to point out plaque accumulation. The model was compared with a trained pediatric dentist and achieved clinically acceptable performance levels. However, there are certain limitations, as the results vary greatly depending on the resulting image’s accuracy, and the underlying logic behind the AI’s plaque identification tactics remains unknown [21]. Once these limitations are cleared, AI technology can be adopted not only by clinicians but also by parents to keep a check on their kids’ oral hygiene in day-to-day life.

### 4.2. Assessing Children’s Oral Health Using Toolkits Designed by Machine Learning

In general, mankind does not give much importance to oral health in comparison to other parts of the body, and not even a majority of the population gets an annual oral checkup. This is especially true in underdeveloped and developing countries. The World Health Organization (WHO) developed an oral health questionnaire [20] for all adults and children to address these concerns. A research team aimed to make use of machine learning to build oral health assessment toolkits that was well equipped to predict the Children’s Oral Health Status Index (COHSI) and Referral for Treatment Needs (RFTN) [20,47]. Liu et al. [47], in their study, prepared a conceptual model (oral health item bank system) guided by the PROMIS framework. The oral health conceptual model was developed by an expert panel of pediatric dentists, general dentists, social scientists, and PROMIS experts. The conceptual model is divided into three main components: physical, mental, and social health. The oral health item bank system created in this paper provides the foundation for other purposes, such as creating specific targeted short forms for program evaluation and/or oral health policy planning, and others. Wang, Y. et al. [20] developed a tool kit which consisted of a short form (SF) to assist parents in evaluating their children’s oral health status and need for treatment, which conceptualized health as having physical, mental, and social components. The toolkit’s (SF) accuracy greatly depended on the way questions were framed, the knowledge quotient of kids and their parents, the time of day when the survey was done, and, above all, how the machine learning algorithm was developed [20]. The main idea of this toolkit was to additionally help the dentist with dental examinations, but never as a total replacement for the physical oral check. The toolkit came up with treatment needs for participants, if needed, predicted overall oral health status using the COHSI score, and generated ranks by the percentile among them [20].

A study by Gajic et al. [7] analyzed the impact of oral health on adolescent quality of life using statistical methods and artificial intelligence algorithms and found that human intuition and machine algorithms both agreed on how the responses should be split. The respondents may be divided into distinctive groups using artificial intelligence algorithms, allowing the finding of information not available with the intuitive classification of respondents by gender.

The machine learning-based toolkit results could be used by all—dentists, parents, and even kids to understand an individual’s need for oral treatment and to get an idea where someone stands in terms of oral health. It will be necessary for dental education to complement the introduction of clinical AI solutions by encouraging digital literacy among those who will work in the dental field in the future. Hence, incorporating machine learning in dentistry is highly beneficial, as we can achieve rapid results with better accuracy [48,49].

### 4.3. Mesiodens and Supernumerary Tooth Identification

Artificial intelligence finds its use in diagnosing mesiodens by utilizing single deep learning models [23]. Missing the presence of supernumerary teeth on panoramic radiographs is largely due to the screening performance of young and inexperienced dental personnel [50]. Additionally, not many general dentists are versatile in diagnosing mixed dentition in children. With such disadvantages, CNN-based deep learning could provide extensive support in screening supernumerary teeth [3]. Ahn, Y. et al. [23] used a deep learning model to detect mesiodens in primary or mixed dentition, implying that this method could help clinicians with limited clinical experience accomplish more accurate and timely diagnoses. They made use of multiple deep learning models (Squeeze net, ResNet 18, ResNet 101, and Inception-ResNet-V2) with the simple correlation that deeper networks provide better accuracy for classifying mesiodens. Two deep learning models were notably faster in coming up with results compared to human evaluation, but their accuracy rate was slightly lower compared to human detection, which was significantly faster.

Interestingly, a retrospective study by Mine, Y. et al. [3] made use of three CNN models (AlexNet, VGG16-TL, and InceptionV3-TL) to single out the presence of supernumerary teeth in the early mixed dentition stage, and surprisingly, all three models performed well. These models have the benefit of being simple enough to be used in a clinical setting without too much difficulty. There are a couple of disadvantages, including the limited availability of datasets and AI-based models that are capable of labeling even those images that are out of reach for two-dimensional panoramic radiographs. In order to improve their performance to a level that is more applicable in the real world, their training must include a large number of medical photos that have been obtained from multiple facilities/institutions. There are a couple of disadvantages, including the limited availability of datasets from a single institution, but AI-based models are capable of labeling even those images that are out of reach for two-dimensional panoramic radiographs [3].

According to the findings of the research carried out by Kuwada et al. [22], deep-learning algorithms (DetecNet and AlexNet) have the potential to detect maxillary impacted supernumerary teeth on panoramic radiographs. However, they also noted that this detection is challenging due to the existence of unerupted permanent teeth in the patient’s mouth. In order to identify mesiodens in panoramic radiographs of primary, mixed, and permanent dentition groups (primary, mixed, and permanent dentition), Ha et al. [24] suggested a model based on YOLOv3, and they showed that this model was effective in clinical practice to detect mesiodens on panoramic radiographs of all dentition types when compared to the study done on permanent dentition by Kuwada et al. [22]. The YOLO method is a prominent example of a deep learning (DL) detection technique, and it has shown much superior performance when compared to other detection algorithms. The smaller sample size was also a limitation of their study, and it was suggested that a larger sample size from more centers would improve the model’s performance. Research conducted by Kaya, E. et al. [10] evaluated the effectiveness of a deep learning system for detecting permanent tooth germs, and the authors concluded that a deep learning-based technique might aid in the early identification of dental deficiencies or supernumerary teeth. It was also hypothesized that dentists may save time and energy while accessing better precise treatment alternatives. Kim, J. et al. [41] employed a deep learning system [DeeplabV3 plus and Inception-resnet-v2] to diagnose mesiodens and observed that a completely automated procedure for identification was possible but that the number and position could not be established.

Hence, the CNN-based deep learning method is a promising technology for supporting dentists in their diagnostic work; nonetheless, more improvements are required for clinical applications before it can be used. In the not-too-distant future, it will be required to construct an all-encompassing diagnostic system that can accommodate a greater variety of ages and conditions. As a result, the use of CNN-based deep learning may improve screening by non-pediatric dentists and allow pediatric dentists to develop treatment plans at an earlier stage.

### 4.4. Early Childhood Caries

There are several factors associated with ECC, thereby making it a multifactorial problem [25,51]. The factors responsible seem disconnected from the environmental and behavioral factors, making us wonder whether there is an underlying biological factor, i.e., genetic factor, that imposes a greater effect on caries formation [26]. Researchers have come up with different genes and gene polymorphisms responsible for dental lesions in patients, but most studies lack genetic factors associated with the disease [26,52]. Making use of single nucleotide polymorphisms (SNPs) for predicting the risk of dental caries could be a highly valuable tool for clinicians to accommodate prevention strategies during the early stages of a child’s life and for parents in terms of inculcating improved eating habits, according to Zaorska, K. et al. [26]. In their study, the researchers made use of artificial neural networks to predict the presence of dental caries based on polymorphisms. The data from such predictions could help avoid caries altogether in children by taking appropriate measures, incorporating early treatments for affected caries, and, thereby, improving the child’s overall quality of life.

Machine leaning-based models (XG Boat, random forest, and light GBM) were compared with a regression model for detecting early childhood caries in the study by Park, Y.H. et al. [29] Though the team used three ML algorithms to come up with a prediction model and checked the results against a logistic regression model, no strong differences were observed. With its own set of limitations, this model helps in predicting the presence of ECC in preschool children with the aid of simple surveys and examinations. It is possible to utilize the model to determine which groups are at high risk for ECC, to carry out active preventive therapies, and to formulate policies on ECC prevention. Our goals are to increase the positive impacts of oral health education on the guardians of preschool children and to contribute to a decrease in the occurrence of ECC.

Koopaie, M. et al. [27] used statistical analysis and machine learning approaches to compare the levels of salivary cystatin S and demographic data between ECC patients with caries-free patients. Different types of supervised learning models, including feed-forward neural networks, XGBoost, Random Forest, and Support Vector Machines (SVM), were used in this study. The results of their study suggested that the effectiveness of machine learning approaches to discriminate early childhood caries from caries-free controls could be improved by utilizing salivary cystatin S levels. The use of machine learning techniques does not make it simpler to discover key elements in assessing ECC levels; rather, it assists us in the development of computer algorithms that are able to take into account a collection of variables and the complex interactions between them. The field of dentistry might benefit from using machine learning as a screening tool.

Pang, L. et al. [28] conducted research to develop a new caries risk prediction model (CRPM) that took into account both environmental and genetic factors. At the community level, CRPM can be used to identify high-caries-risk populations, allowing policymakers to plan necessary preventative actions for the future.

Karhade, D. S. et al. [29] developed and evaluated an automated ML application for ECC-based child classification. The results of the study suggested that a parsimonious model had the highest classification performance. A relatively naïve machine learning model based on children’s age and parental opinion of oral health may predict ECC risk, and machine learning can create high-quality classifiers that can infer ECC status from proxy-reported and demographic data.

With the advent of COVID-19, many individuals prefer online consultations, and in such cases, it becomes mandatory for parents to have better knowledge and ideas about oral health, as well as concrete ways to assess their kid’s dental hygiene and convey the oral health status to the clinician [30]. In such times, having a definite list of survey questions helps in assessing the kid’s oral health when a physical assessment by the dentist is impossible. To come up with the best set of questions, a study by Ramos-Gomez, F. et al. [30] came up with the idea to make use of an ML algorithm named random forest (RF) that picks the top-most questions from the parent’s questionnaire that can predict the presence of active caries. Using these findings, the team examined the study participants physically. So, machine learning algorithms based on oral health surveys may assist dentists in anticipating dental caries in newborns and young children. Dental clinicians may incorporate the primary predictors of dental caries in their caries risk assessment and educate patients and caregivers about excellent oral hygiene.

ML has been used for designing models that have become excellent tools for physicians, helping them with decision-making skills and being used in various public-related health applications. However, the use of ML in oral care is not widely used in conjunction with ECC [53].

### 4.5. Fissure Sealant Categorization

Dental sealants are widely used as a protective coating on the chewing surfaces of molars to protect them from cavities [54]. For every type of dental problem present, there are different interventions, including dental restorations, sealants, and prosthodontic measures. Convolutional neural networks (CNNs) are used profoundly to classify diagnostic images and objectify pathological findings’ classification, but these networks must be trained exclusively to identify each of the problems [31]. CNN is an integral deep learning algorithm that relies on heaps of data to assist dental practitioners. Additionally, dental sealants are the first go-to solution for many dental problems and can be easily identified as they are generally white in color [31]. Hence, fine-tuning CNN to identify dental sealants seems to be the most logical solution. A research team led by Schlickenrieder, A. et al. [31] developed a deep learning-based CNN to identify these sealants from machine-readable intraoral photographs. In comparison, this AI-based algorithm delivered a high diagnostic accuracy compared to the normal CNN-based classifications. However, there were a couple of limitations that needed in-depth dental research, repeated training for accurate detection, and categorization of the various diseases and their restoration procedures before using this AI-trained CNN in clinical applications.

### 4.6. Chronological Age Assessment in Kids and Adolescents Using Neural Modeling

For clinicians, analyzing the age of excavated or forensic human remains to make the best treatment choices, as well as assessing the age of children during adoptions or illegal stays in some countries, makes knowing the metric age assessment indispensable [32]. Teeth development is quicker in girls compared to boys, largely due to sexual dimorphism. Implementing artificial neural networks to handle medical-related data has gained prominence in recent times, and it also offers better and more efficient diagnostics in various medical conditions [32,55,56].

Dental age is generally analyzed using one of two methods, namely the clinical method or the pantomographic method. While the clinical method is easy to apply and gives results quickly, it produces highly inaccurate outcomes. On the other hand, pantomographic methods of assessment that assess the mineralization of tooth buds are more precise [32]. There have been several methods formulated until now, each of which could be used on children and adolescents of different ages and displayed different accuracy levels.

Zaborowicz, K. et al. [32] focused on producing a new method for detecting the chronological age of kids and adolescents aged 4–15 years using digital pantomographic images and neural modeling. This method is simpler, has a near-perfect accuracy, and was one of the first to use pantomographic images for metric age assessment; however, one of its major limitations is that it does not use 2D photographs and only works with pantomographic images [32].

A study by Zaborowicz, M. et al. [37] used 3 deep neural network models for assessing the chronological age of kids and adolescents aged 4–15 years and showed that neural modeling algorithms may accurately determine metric age using proprietary teeth and bone indicators. Bunyarit, S.S. et al. [33] used an artificial neural network (ANN) computational technique to create new dental maturity ratings based on Demirjian’s scores in their study. They observed that the new dental maturity scores may determine the age of Malaysian Chinese children and adolescents.

Lee, Y.H. et al. [38] conducted an interesting study that used 18 radiomorphometric parameters extracted from panoramic radiographs (PRs) and focused primarily on developing ML algorithms. They observed that ML algorithms are more efficient at estimating age compared to traditional estimation.

### 4.7. Detecting Deciduous and Young Permanent Tooth

CNN, one of the most popular architectures of deep learning, is commonly used for object recognition. Deep learning methods such as CNN are increasingly being used for assessing and enumerating deciduous teeth in pediatric patients [34]. For the purposes of object identification and detection, a number of models, such as R-CNN, Faster R-CNN, YOLOv3, and YOLOv4, have been used. Object detection techniques are divided into two categories: single-stage detectors (YOLO algorithm) and two-stage detectors (Mask-RCNN, R-CNN, and Faster R-CNN) [57]. Tooth identification serves as the foundation for automated and complex detection systems, which subsequently identify which teeth are affected by dental diseases and assign those disorders to the identified teeth. Researchers have come up with various techniques to classify and number the teeth and moved on to region-based and threshold techniques for numbering the teeth in the last decade or so, as opposed to CNN-based classification [34]. However, CNN-based mapping has shown superior accuracy in the field of automated tooth segmentation [58]. The use of panoramic radiographs to number primary teeth is one step closer to achieving digital diagnostic solutions that reduce not only time but also significant effort in the field of dentistry [34].

CNN algorithms were employed by Caliskan, S. et al. [35] to identify and categorize submerged molars, and the researchers found that the approach was effective. Using tooth-numbering algorithms to identify the absence of a certain tooth germ requires further studies. Dental practitioners may benefit from the identification of missing tooth germs in order to determine more accurate treatment techniques. Kilic, M.C. et al. [34] investigated a faster R-CNN inception v2 approach for recognizing and numbering primary teeth on pediatric panoramic radiographs, and they reported good sensitivity and accuracy scores. They noticed from their study that only primary teeth were detected and numbered, which also plays a valuable role in forensic identification.

Kaya, E. et al. [39] tested the effectiveness of a deep learning system for automated tooth recognition and counting using YOLOv4, a CNN-based object identification model. The model was able to recognize and count both primary and permanent teeth. The YOLOv4 method is a well-known one-stage detector model that can identify and categorize objects in a single image. This model is a real-time object identification system that identifies several things and draws bounding boxes around each detected object to represent the area of detection. YOLOv4 was employed for object recognition because of its exceptional speed and precision.

Some research have employed two-stage detectors and produced excellent object detection results. Two-stage detectors are often more accurate than one-stage detectors, but they are also more time-consuming and computationally intensive. Therefore, YOLO is an example of a single-stage detector utilized for fast and accurate object identification and categorization. YOLO stands out from other CNN algorithms because of its ability to do real-time object recognition while also achieving above-average results over a wide range of object classes on average [39].

### 4.8. Ectopic Eruption of First Permanent Molar

When a tooth erupts in an irregular location, called an “ectopic eruption”, it often occurs during early mixed dentition. The maxillary first molar is the most commonly missing tooth. Possible side effects include narrowing of the dental arch, loss of interdental space, malocclusion, and absorption of the distal surface of the primary second molar [59]. Hence, an early diagnosis may aid in treatment planning and perhaps avert unwanted problems [60]. Clinical and radiological evaluations are used to identify ectopic eruptions. There are many types of radiographs used in the diagnostic process, including cone beam computed tomography (CBCT), panoramic, occlusal, and periapical radiography. All these panoramic radiographs analyze all teeth and surrounding structures, determining tooth position and ectopic eruption [36]. Nevertheless, 2D imaging, restricted magnification, and anatomical structural superposition are drawbacks of panoramic radiography. Radiomics and AI have advanced rapidly. Multi-layered CNNs help dentists diagnose more precisely, consistently, and comprehensively. Zhu, H. et al. [36] found that the model (nnU-Net) was more consistent and accurate in detecting and segmenting ectopic eruptions in molars in the mixed dentition period. They did a performance analysis on various models, including U-Net, R2U-Net, attention U-Net, and nnU-Net, and discovered that nnU-Net had the best results when it came to semantic augmentation. Semantic segmentation is difficult, and algorithm performance depends on dataset complexity and size [61]. By modifying features, such as data processing and training techniques, no-new-Net (nnU-Net) can dynamically adapt to any dataset and produce optimum model impact [35,62,63]. Liu, J. et al. [40] developed an automated screening approach that can identify ectopic maxillary molar eruption with accuracy comparable to pedodontists. The authors found that AI-assisted image recognition models may improve the accuracy of human interpreters; however, deep learning is still not 100% accurate for using them for identification.

Some of the shortcomings of this paper might be related to the search approach. Despite a thorough search conducted for the required articles, some might have been missed out. Regardless of the benefits, AI remains undesirable for a variety of reasons. The absence of general and methodical standards for AI development, as well as the lack of complete data, accessibility, structure, and comprehensiveness, and the difficulties associated with acquiring them [4], were the key limitations impeding its efficacy. Nonetheless, more questions are being raised about the ethics, responsibilities, worth, and use of artificial intelligence in human existence. The difficulties associated with maintaining patients’ anonymity as well as the reluctance of dental professionals to use AI-based procedures considering the need to maintain human engagement in clinical care were the primary causes of discontentment among dental professionals.

However, given the performance of these AI models, there is an urgent need to establish and execute rules to expedite the process of promoting and employing these models in clinical settings, which may assist professionals in diagnosing and determining an appropriate treatment strategy.

## 5. Conclusions

Now, without a doubt, we can say that AI can never be a replacement for dental clinicians/pediatric dentists but can be of assistance in every area of dental health, including preventive dentistry, restorative dentistry, diagnostic sciences, and more. AI has been routinely used in pediatric dentistry to assist less experienced dentists in making more accurate diagnoses. These models are of great help at the individual and community levels and are effective in identifying and categorizing children into risk groups, identifying, and numbering the tooth, diagnosing early ectopic eruption, age assessment, etc. They can also be used as an aid in the planning and assessment of school oral health programs, thus making the children more aware of their own dental health. As a complementary aid, AI can be used in a controlled way ensuring that the human touch is not lost and reinstating the fact that dentists/pediatric dentists hold the reigns to control treatment protocols and take decisions. However, the day when dental practitioners and AI might collaborate for better patient care is not far away.

## Figures and Tables

**Table 1:** Details of the studies that reported AI-based models in pediatric dentistry.

Sl No	Author	Year	Algorithm Architecture	Objective	Outcome	Authors Observation
1	Wang, Y. et al. [20]	2020	ANNs	To build oral health assessment toolkits to predict children’s oral health status index [COHSI] score and referral for treatment needs of oral health [RFTN]	The sensitivity and specificity for predicting RFTN were 93% and 49%. The RMSEs of the COHSI tool kit were 4.2 for COHSI.	The tool kits can be used by oral health programs and also to quantify differences between pre- and post-dental care program implementation. The tool kits can be used in oral health research for stratifying samples.
2	You, W. et al. [21]	2020	CNNs	To analyze the accuracy of an AI-based model for detecting plaque on primary teeth	Significant difference was not observed for AI model and specialist. A CNN-based model demonstrated high accuracy in detecting plaque in comparison with the pediatric dentist.	AI model will definitely help children in appreciating and improving their oral health.
3	Kuwada, C. et al. [22]	2020	CNNs	To compare the performance of 3 deep learning systems for classifying maxillary impacted supernumerary teeth in patients with fully erupted incisors	Three learning models AlexNet, VGG-16 and DetectNet were used. Detect net Produced highest values of diagnostic efficacy.	The models have potential use in classifying the presence of impacted supernumerary teeth in the maxillary incisor region.
4	Bunyarit, S. S. et al. [33]	2020	ANNs	To develop reliable teeth maturity scores for age estimation based on artificial neural networks	Significant correlation was observed between chronological. Age and new dental maturity scores after ANN in both girls and 20 boys (*p* < 0.001) with greater accuracy in age estimation.	Can be applied to clinical and forensic cases.
5	Gajic, M. et al. [7]	2021	ANNs	To determine the impact of oral health on adolescent’s quality of life	Respondents can be clustered into characteristic groups using AI.	Introduction of clinical AI solutions in dental education will foster digital literacy in the future dental workforce.
6	Ahn, Y. et al. [23]	2021	CNNs	To classify mesiodens in primary or mixed dentition	Greater accuracy was observed in classifying the presence of mesiodens in the mixed dentition panoramic radiographs using AI models.	More accurate and faster diagnosis was made possible through deep learning technologies.
7	Ha, E. G. et al. [24]	2021	CNNs	To develop an artificial intelligence model to detect mesiodens on panoramic radiographs in all dentition types	A CNN model based on YOLOv3 showed good performance in all dentition types.	The models have better use in detecting mesiodens.
8	Park, Y.H. et al. [25]	2021	ANNs	To predict early childhood caries using ML-based AI models (XG Boost, random forest, Light GBM algorithms and Final model)	ML-based models showed favorable performance in predicting DC with a satisfactory AUC value and no significant difference between AUROC values of 4 models.	Can be useful in identifying high-risk groups and implementing preventive treatments.
9	Zaorska, K. et al. [26]	2021	CNNs	AI model for predicting DC based on chosen polymorphisms	This model displayed high accuracy in predicting DC with sensitivity of 90, specificity of 96% and overall accuracy of 93% and AUC of 0.97.	The knowledge of potential risk status could be useful in designing oral hygiene and adopting healthy eating habits for patients.
10	Koopaie, M. et al. [27]	2021	ANNs	To detect the salivary level of cystatin S in ECC patients and caries-free (CF) children	The logistic regression model based on salivary cystatin S levels and birth weight had the most acceptable potential for discriminating early childhood caries from caries-free controls.	Considering clinical examination, demographic and socioeconomic factors, along with the salivary cystatin S levels could be useful for early diagnosis of ECC.
11	Pang, L. et al. [28]	2021	ANNs	To predict caries risk based on environmental and genetic factors	This model could accurately identify individuals at high and very high caries risk.	This is a powerful tool for identifying individuals at high caries risk at the community level.
12	Karhade, D. S. et al. [29]	2021	ANNs	To evaluate the accuracy of an automated ML algorithm for classification of early childhood caries (ECC)	This ML model’s performance was similar to the reference model with AUC of (0.74), sensitivity of (0.67), and PPV of (0.64).	This model is valuable for ECC screening.
13	Ramos-Gomez, F. et al. [30]	2021	ANNs	To identify survey items for predicting dental caries (DC)	Development of algorithm “toolkits” that help dental professionals assess their patient’s oral health could prove extremely useful for prevention of dental caries among children.	This model has potential for screening DC.
14	Schlickenrieder, A. et al. [31]	2021	CNNs	To assess the performance of convolutional neural network (CNN) for detecting and categorizing fissure sealants	CNN detected sealant intraoral photographs with an agreement of 98.7% in comparison with reference decisions.	Additional training in AI-based techniques is required before clinical use.
15	Zaborowicz, K. et al. [32]	2021	ANNs	To estimate age using Deep learning based models	This newly developed methodology may serve as an algorithm for implementation in a computer application, which will automatically determine the chronological age of children and adolescents between the ages of 4 and 15.	It is possible to develop a new methodology for the assessment of chronological age on the basis of digital pantomographic images with the determination of a new set of tooth and bone parameters.
16	Kilic, M. C. et al. [34]	2021	ANNs	To evaluate use of deep learning approach for automated detection and numbering of deciduous teeth in children on panoramic radiographs	The faster R-CNN inception v2 models were successful in detecting and numbering the deciduous teeth of children with higher sensitivity and precision rates.	Deep learning-based AI models are a promising tool for the automated charting of panoramic dental radiographs.
17	Caliskan, S. et al. [35]	2021	CNNs	To compare the success and reliability of AI applications in the detection and classification of submerged teeth in panoramic radiographs	The detection model, Faster R-CNN was extremely accurate in its performance.	Useful diagnose submerged molars with an AI application to prevent errors and facilitate the diagnosis of pediatric dentists.
18	Zhu, H. et al. [36]	2022	CNNs	To automatically segment and detect ectopic eruption of first permanent molars in early mixed dentition on paranoic radiographs	The study revealed that the AI model was statistically significant and superior in terms of IoU, precision, F1 score, accuracy, FROC, and reliability.	This research validated a nnU-Net-based AI model for segmenting and identifying Ectopic eruption of permanent maxillary molars on panoramic radiography.
19	Mine, Y. et al. [3]	2022	CNNs	To detect the presence of supernumerary teeth during the early mixed dentition stage	All the models demonstrated high accuracy with higher sensitivity and specificity values. VGG16-TL model had the highest performance in comparison with others.	CNN-based deep learning is a promising method for detecting the presence of supernumerary teeth during the early mixed dentition stage.
20	Kaya, E. et al. [10]	2022	CNNs	To assess the performance of a deep learning system for permanent tooth germ detection on pediatric panoramic radiographs.	The YOLOv4 model, which detected permanent tooth germs on pediatric panoramic radiographs, provided an average precision value of 94.16% and an F1 value of 0.90, indicating a high level of significance.	Can be applied for early diagnosis of tooth deficiency or supernumerary teeth by detecting of permanent tooth germs and also helpful in planning more accurate treatment options while saving time and effort.
21	Zaborowicz, M. et al. [37]	2022	CNNs	To estimate age using deep learning-based models compared to models used previously	The MAE error of the produced models, depending on the learning set used, was between 2.34 and 4.61 months, while the RMSE error was between 5.58 and 7.49 months. The correlation coefficient R^2^ ranged from 0.92 to 0.96.	The study reveals that neural modeling approaches are an acceptable tool for predicting metric age based on proprietary tooth and bone indices created.
22	Lee, Y.H. et al. [38]	2022	MLs	To investigate the relationship of 18 radiographic parameters of panoramic radiographs based on age, and to estimate the age group of people with permanent dentition using five machine learning algorithms	The area under the curve (AUCs) obtained for classifying young ages (10–19 years) ranged from 0.85 to 0.88 for five different machine learning models.	Using numerous maxillary and mandibular radiomorphometric data, they developed suitable linear and nonlinear machine learning models for estimating dental age groups.
23	Kaya, E. et al. [39]	2022	CNNs	To evaluate the performance of a deep learning system for automated tooth detection and numbering on pediatric panoramic radiographs	The proposed CNN YOLOv4 method yielded high and fast performance for automated tooth detection and numbering on pediatric panoramic radiographs with the mean average precision (mAP) value of 92.22%, mean average recall (mAR) value of 94.44%, and weighted F1 score of 0.91.	Automatic tooth detection could help dental practitioners to save time and also use it as a pre-processing tool for detection of dental pathologies.
24	Liu, J. et al. [40]	2022	CNNs	To detect eruption of maxillary first molar in panoramic radiographs aged 4–9 years old by using a semi-automated fusion model	Deep learning-based automated screening for maxillary Permanent First Molar is promising and showed higher sensitivity and specificity.	A semi-automatic screening algorithm may enhance clinical diagnosis of Ectopic eruption over human specialists and help in management.
25	Kim, J. et al. [41]	2022	CNNs	To develop and evaluate the performance of deep learning-based identification of mesiodens in panoramic radiography	The categorization of mesiodens might potentially be fully automated according to the deep learning system’s findings with respect to accuracy, IoU, precision, and F1 score.	The posterior molar space on the panoramic radiograph served as the foundation for the deep learning system’s diagnostic performance, which will be used to automatically diagnose various disorders using just panoramic radiographs.

Foot notes: MLs = Machine Learning ANNs = artificial neural networks, CNNs = convolutional neural networks, MAE = mean squared error, RMSE = Root mean squared Error, RR = Relative risk, AUC = Area under the curve, mAP = mean average Precision, mAR = mean average at recall, IoU = Intersection over union, PPV = Positive Predictive Value, AUROC = Area under the receiver operating characteristic, FROC = Area under the free response ROC curve study.

**Table 2:** Summary of AI models designed for different diagnostic tasks.

AI Technique/Algorithm Architecture	Diagnostic Tasks	Functionality of the AI Model	Input Features
Machine Learning (ML)	Automated estimation of the age	Landmarks	Radiomorphometric data [Lee, Y.H. et al.] [38]
Artificial Neural Networks (ANNs)	For predicting children’s oral health status index (COHSI) and referral for treatment needs [RFTN]	Oral health status	Data sets [Wang, Y. et al.] [20]
	Classification of early childhood caries	Dental caries	Data sets [Karhade, D.S. et al.] [29]
	Determining the chronological age	Age assessment	Digital pantomographic images [Zaborowicz, K. et al.] [32]
	Caries risk prediction	Dental caries	Data sets [Pang, L. et al.] [28] [ Ramos-Gomez, F. et al.] [30]
	Predicting early childhood caries	Dental caries	Data sets [Park, Y.H. et al.] [25] [Koopaie, M. et al.] [27]
	Impact of oral health on adolescents’ quality of life	Adolescent quality of life	Data sets [Gajic, M. et al.] [7]
	Age estimation	Dental age and Chronological age	Panoramic images [Bunyarit, S.S. et al.] [33]
Deep Learning (CNNs)	Detecting plaque on primary teeth	Dental Plaque	Intra oral photographs [You, W. et al.] [21]
	Detecting and categorizing fissure sealants	Fissure sealants	Digital photographs [Schlickenrieder, A. et al.] [31]
	Predicting dental caries based on chosen polymorphisms	Dental caries	Data sets [Zaorska, K. et al.] [26]
	Detection and enumeration of the deciduous teeth	Tooth	Panoramic images [Kılıc, M.C. et al.] [34]
	Automatically classify mesiodens. in primary or mixed dentition	Mesiodens	Panoramic radiographs [Ahn, Y. et al.] [23]
	Identification of mesiodens in growing children/various dentition groups	Mesiodens	Panoramic radiographs [Kim, J. et al.] [41] [Ha, E.G. et al.] [24]
	Detecting the presence of supernumerary teeth during the early mixed dentition stage	Supernumerary teeth	Panoramic radiographs [Mine, Y. et al.] [3]
	Classifying maxillary impacted supernumerary teeth	Supernumerary teeth	Panoramic radiographs [Kuwada, C. et al.] [22]
	Estimating the age	Dental age	Digital Pantomographs [Zaborowicz, M. et al.] [37]
	Automated tooth detection and numbering	Tooth detection	Panoramic images [Kaya E et al.] [10] [Kaya E et al.] [39]
	Detection and classification of submerged teeth	Tooth detection [Submerged teeth]	Panoramic radiographs [Caliskan S et al.] [35]
	Detection of ectopic eruption of permanent maxillary molar	Ectopic eruption	Panoramic radiographs [Zhu H et al.] [36] [Liu J] [40]

## Data Availability

Not applicable.
